# Associations of Sleepiness Scores and Cognitive Performance in Cognitively Normal Non‐Sleepy Older Adults – Examining for Race and Sex‐specific Differences

**DOI:** 10.1002/alz70858_107453

**Published:** 2025-12-26

**Authors:** Rachel A McCray, Anthony Q Briggs, Mark A Bernard, Joshua L Gills, Elena Valkanova, Mobeena Ghuman, Allal Boutajangout, Ludovic Debure, Girardin A Jean‐Louis, Arjun V. Masurkar, Ricardo S. Osorio, Omonigho M Bubu

**Affiliations:** ^1^ NYU Grossman School of Medicine, New York, NY, USA; ^2^ University of South Florida, Tampa, FL, USA; ^3^ University of Miami Miller School of Medicine, Miami, FL, USA

## Abstract

**Background:**

Excessive Daytime Sleepiness (EDS) may impair cognitive performance and increase Alzheimer's Disease risk. However, its impact across race and sex in non‐sleepy older adults is understudied. We examined whether sleepiness scores were linked to cognitive performance and if this association varied by race and sex.

**Method:**

We conducted a cross‐sectional analysis of data from 186 cognitively normal community‐dwelling healthy older‐adults (*n* = 103 whites, *n* = 83 blacks) participating in various NYU studies on sleep, aging, and memory. Sleepiness and cognitive performance were characterized using the Epworth Sleepiness Scale (ESS) and Uniform Data Set (UDS) Neuropsychological Battery, respectively. Plasma AD biomarkers were measured inclusive ptau181, and aβ42. Linear mixed effects models controlling for age, sex, race, education, aβ42, and ptau181, examined the association of sleepiness scores with cognitive performance across memory, visuospatial, attention, language, processing speed, and executive function domains. The effects of ESS*race and ESS*sex on cognitive performance were also examined.

**Result:**

Average age of participants was 72.7±6.5 years, with 16.6±2.2 years of education, 68.8% of participants were female and 44.6% Black, with an average ESS of 4.2±3.7, and 407 ±85.6 minutes of total sleep time. ESS showed no independent effect on cognitive performance (*p* > .05). Race was associated with cognitive performance, with Blacks performing worse than Whites (*p* < .05). Sex was associated with performance in memory and executive functioning tasks, respectively (β[Sex]=2.10 [0.14, 4.05]; β[Sex] = ‐46.15 [‐85.90, ‐6.40]; *p* <0.05) with females performing better in memory tasks but worse in executive function tasks compared to males. ESS's relation with cognitive performance differed between races in memory and language tasks. (β[Race *ESS] = 0.54 [0.05,1.04]; β[Race*ESS] = 0.36 [0.05, 0.67]; *p* <0.05), with Blacks reporting more sleepiness performing worse. No interaction effects were found between sex and ESS on cognitive performance.

**Conclusion:**

In this non‐sleepy, cognitively healthy diverse sample of community dwelling older adults, sleepiness scores were not associated with performance in any cognitive domain, possibly because this was a non‐sleepy cohort. However, Race‐specific effects of this association were observed in cognitive domains that subserve memory and language functions, suggesting that impacted domains may be related to EDS symptoms with a notable effect among Blacks.

